# Salivary Oxytocin Concentrations in Seven Boys with Autism Spectrum Disorder Received Massage from Their Mothers: A Pilot Study

**DOI:** 10.3389/fpsyt.2015.00058

**Published:** 2015-04-21

**Authors:** Shuji Tsuji, Teruko Yuhi, Kazumi Furuhara, Shogo Ohta, Yuto Shimizu, Haruhiro Higashida

**Affiliations:** ^1^Department of Clinical Psychology, School of Psychological Science, Tezukayama University, Nara, Japan; ^2^Department of Basic Research on Social Recognition, Research Center for Child Mental Development, Kanazawa University, Kanazawa, Japan

**Keywords:** autism spectrum disorder, massage, salivary oxytocin, touch therapy, mothers

## Abstract

Seven male children with autism spectrum disorder (ASD), aged 8–12 years, attending special education classrooms for ASD and disabled children, were assigned to receive touch therapy. Their mothers were instructed to provide gentle touch in the massage style of the International Liddle Kidz Association. The mothers gave massages to their child for 20 min every day over a period of 3 months, followed by no massage for 4 months. To assess the biological effects of such touch therapy, saliva was collected before and 20 min after a single session of massage for 20 min from the children and mothers every 3 weeks during the massage period and every 4 weeks during the non-massage period, when they visited a community meeting room. Salivary oxytocin levels were measured using an enzyme immunoassay kit. During the period of massage therapy, the children and mothers exhibited higher oxytocin concentrations compared to those during the non-massage period. The changes in oxytocin levels before and after a single massage session were not significantly changed in children and mothers. The results suggested that the ASD children (massage receivers) and their mothers (massage givers) show touch therapy-dependent changes in salivary oxytocin concentrations.

## Introduction

Sensory functioning is recognized to be different for children with autism spectrum disorder (ASD) (1, 2). Sensory impairment may lead to a dislike of going outside, washing the hands and body, and dressing. They may display tactile defensiveness in the form of rubbing, scratching, self-stimulation such as rocking and self-injury (3). Thus, the role of the senses for children with ASD is an important aspect when considering means of improving quality of life for these patients (4, 5). However, little biological evidence is available regarding the role of the senses in ASD (6).

Physical contact is one of the major communicative interactions to form attachment among normal parents and children (7, 8). In contrast, parents and children with ASD may be aversive to and avoidant of touch (9). However, the use of predictable touch in the form of massage has been shown to decrease touch aversion and reduce stereotypic behaviors in children with ASD (10–14). With the predictable movements of massage, this type of touch may be more acceptable than the unpredictable social touch frequently resisted by children. In addition, light rather than deep touch and longer intervention rather than short periods of massage seem to be efficient (15).

Recent studies suggested that oxytocin (OT) plays a role in social behavior (16–22). OT was shown to have positive effects on social and emotional processes in both healthy subjects and individuals diagnosed with a variety of psychiatric disorders (9, 23, 24). Some previous reports indicated positive or negative relations of increases in blood OT concentrations associated with improvement of human behaviors after massage (25, 26). However, there have been few reports regarding the effects of touch therapy in ASD children.

Salivary OT can be measured and intranasal OT administration is reflected in human saliva, suggesting that it may be a reliable biomarker (27–34). Therefore, we examined whether male school children with ASD exhibit increases in OT concentration with touch therapy intervention. In this pilot study, the mothers of seven school children with ASD applied gentle touch massage according to the International Liddle Kidz Association style, designed by Tina Allen at the University of California Los Angels (http://www.liddlekidz.com/about-tina-allen.html), every day for 3 months. We measured salivary OT in the children (massage receivers), who received the massage from their mothers, and in their mothers (massage givers).

## Materials and Methods

### Participants

Seven Japanese male children with ASD, aged 8–12 years (10.0 ± 0.49 years), were recruited from special education classes for the disabled and ASD in two elementary schools in Shiga prefecture, Japan. The children had been diagnosed by child psychiatrist at approximately age 2.5–5 years according to the DSM-IV criteria. After being provided a complete explanation of the study, all of the caregivers of the participants provided written informed consent, and each participant provided written informed consent if sufficiently competent to do so. The study was approved by the institutional review board of Clinical Psychology Research in Tezukayama University and by Research Center for Child Mental Development, Kanazawa University.

### Massage therapy

The children received massage therapy from their mothers for 20 min usually after dinner, prior to bedtime, every day for 3 months. Participants’ mothers were trained in massage by a massage therapist (the first author, Shuji Tsuji), who is approved by the Japanese Society for Baby and Child Care and the International Liddle Kidz Society. This involved gentle and warm massage with weak pressure for the child’s body in the following sequence: back, right shoulder to hand, right hand, left shoulder to hand, left hand, and back again.

### Assessment

**Figure 1:**
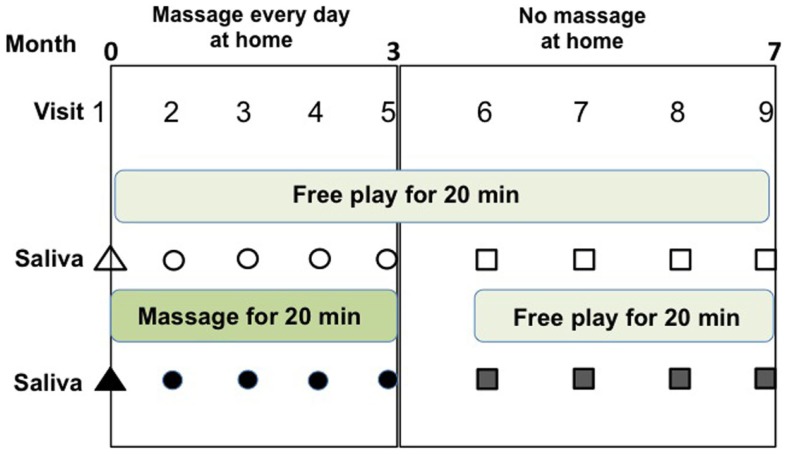
**Schedule of soft touch treatment (massage) and assessment of this study**. Saliva samples were collected at four visits (#2–#5) during the daily massage period for 3 months and four visits (#6–#9) during the non-massage period for 4 months. Saliva was collected before (open symbols) and after (filled symbols) one massage session, or no massage for 20 min during visits to the community center.

The children were assessed for OT levels every 3 weeks during the massage period for 3 months, followed by the first Thursday of each month for 4 months without massage as the control period (Figure 1). The children, mothers, and three authors visited a meeting room in a community house. For the first 20 min, they remained freely (usually children moved and played freely and mothers were sitting and watching their children). Saliva was collected from the children and their mothers in paper cups after washing their mouths. Then, one session of massage was given to the child by his mother for 20 min in a separate room. Saliva was collected a second time after 20 min of resting (free moving play) after massage.

### Saliva collection and analysis

Saliva samples (0.3–0.8 ml) collected in paper cups were immediately transferred to plastic microtubes (1.5 ml). They were frozen and stored at −20°C to be centrifuged twice at 4°C at 1500 × *g* for 15 min 3 days later. The samples were kept at −20°C until assayed.

Determination of salivary OT was performed using a 96-plate commercial OT-ELISA kit (Enzo Life Sciences, Farmingdale, NY, USA), as described previously (31). Recent studies have shown that OT values are reliable when measure in saliva by enzyme immunoassay (30, 31, 34). Measurements were performed in duplicate. Samples (100 μl) were treated according to the manufacturer’s instructions. The optical density of the samples and standards was measured at wavelengths of 405 and 590 nm by a microplate reader (Bio-Rad, Richmond, CA, USA). Sample concentrations were calculated by MatLab-7 according to the relevant standard curve.

### Statistical analysis

The two-tailed Student’s *t*-test was used for single comparisons between two groups. All analyses were conducted using STATA data analysis and statistical software (Stata Corp. LP, College Station, TX, USA).

## Results

**Figure 2:**
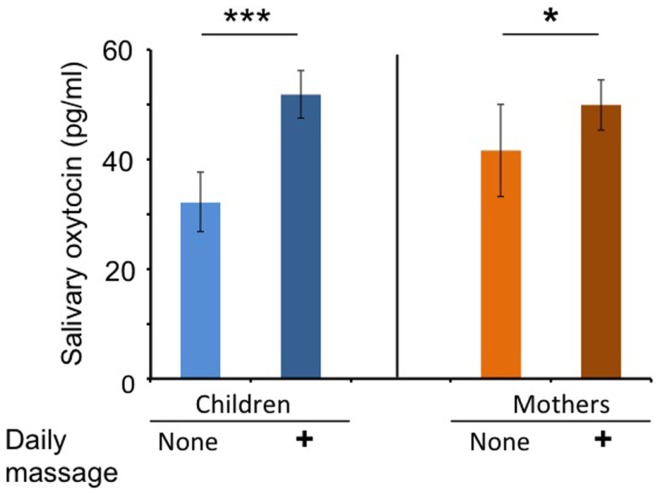
**Oxytocin levels at first saliva collection on each visit during massage or non-massage periods in children and mothers**. Comparison of average OT levels in saliva at baseline (first collection at each visit) for children (massage receivers) and mothers (massage givers) during the daily massage or no massage period. ***P < 0.001; *n* = 53 samples in each period for children. *P < 0.05; *n* = 46 samples in each period for mothers.

The OT concentrations in the saliva collected before each massage session were determined and assigned as the basal OT level. The average basal salivary OT level during the massage period (open circles in Figure 1) was significantly higher than that (open squares) during the non-massage period in children (*n* = 53, two-tailed Student’s *t*-test, *P* < 0.001; Figure 2). Similarly, the average basal OT levels in mothers were higher during the massage period (open circles in Figure 1) than in the non-massage period (open squares) (*n* = 46, two-tailed Student’s *t*-test, *P* < 0.05; Figure 2).

**Figure 3:**
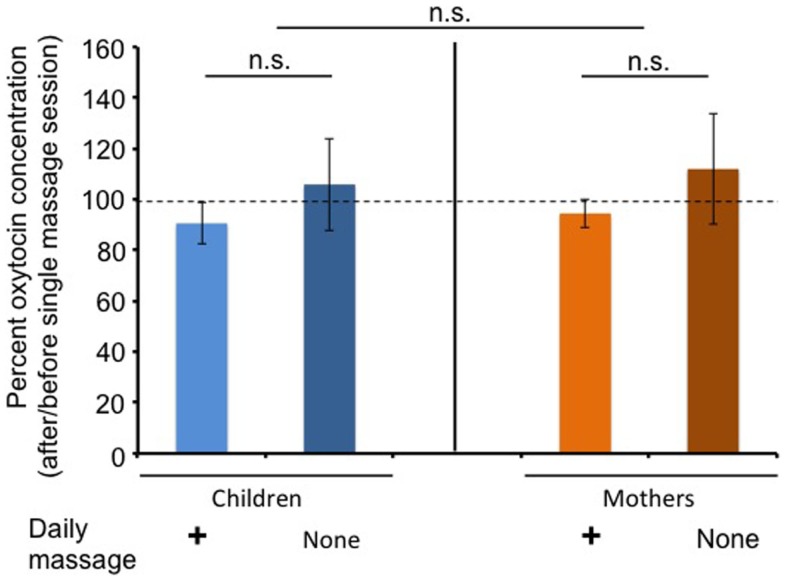
**Changes in average oxytocin levels before and after a single massage session**. The percentage of oxytocin levels at the first (before) saliva over the second (after) saliva. Saliva was collected at each visit during the daily massage or non-massage period in children and mothers.

Figure 3 shows changes in the ratio of salivary OT levels before (open circles) and 20 min after massage for 20 min (filled circles) at massage or free sessions during the massage or non-massage period, respectively. There was no trend that salivary OT levels tended to increase after massage in children and mothers.

## Discussion

We examined the biological effects of touch therapy in children diagnosed with ASD and attending a special education class for ASD and disabled children in elementary schools, as well as in their mothers who provided massage daily. The results indicated that ASD children (massage receivers) and their mothers (massage givers) exhibited higher salivary concentrations of OT during the repeated massage period for 3 months, compared to those during the non-massage (rest) period for 4 months. The effects of current massage appeared in both givers and receivers, which seemed to be due to gentle touch over a long period. This was consistent with a previous report that massage increased OT concentrations in healthy volunteers of university students (26).

Outcome of touch therapy was assessed using a questionnaire asked mothers to indicate their observation of their children’s behaviors upon massage at home. The mother’s feeling was also obtained by interviews before, during, and after massage every day at home. Home Record Sheets were completed by mothers and analyzed. As preliminary results, three children were reported by mothers to be more relaxed. Four children improved reciprocal social interaction and communication to others, but three children were unchanged. There was no report on improved sleep patterns. Surprisingly, the effect of massage by six mothers (85%) was the feeling of closeness to children, as reported (11). Massage appeared to have facilitated the bond between mothers and children that previously may have been disrupted by the nature of autism and by mothers’ inability to understand and communicate with children. Five mothers reported that they were pleased to see their children become relaxed and enjoy receiving massage.

Interestingly, salivary OT concentrations were not increased in children after receiving a single massage session (Figure 3). This was consistent with previous reports (15, 35). This tendency in children was true in the non-massage period. These results suggest that higher OT concentrations during the massage period were not simply due to accumulation with each touch therapy, but due to other as yet unknown effects. Moreover, our results cannot indicate that massage itself seems to be pleasant or stressful for ASD children, who usually have sense impairment.

It has been reported that massage therapy improves classroom performance of children with autism, i.e., reducing off-task behavior and sleep problems (10, 12). The children in the massage therapy group became more attentive at school. This increased attentiveness is similar to the increased on-task behavior noted in the earlier massage therapy study by Field and colleagues (36, 37). The mechanism underlying this enhanced attentiveness is not yet clear. Massage therapy has also been noted to enhance parasympathetic (vagal) activity (38), which is closely correlated with attentiveness (37). The results of the present study, however, suggest that these observations may have been due to increased OT levels.

In humans, there are correlations between peripheral OT and maternal behavior, particularly maternal touch (24, 34, 39). In addition, some single nucleotide polymorphisms on the CD38 genes (16, 23), that mediate the release of OT from hypothalamic neurons into the circulation through the cyclic ADP-ribose-dependent mobilization of calcium, were found to be associated with lower levels of plasma OT (19, 23, 40) and lower levels of parental touch of infants (8). These results and our finding suggest a correlation between touch and OT concentration.

This study had some limitations. Although the clinical test was 7 months in length, the number of school children was small. However, we could obtain meaningful data from the mothers, because they were caregivers of ASD patients, which would not have been possible in simple clinical trials. Unfortunately, we did not perform follow-up searches to check if both mothers and children continue to enjoy giving and receiving touch therapy, respectively. Interviews with mothers and salivary collections for OT concentrations at 24-month follow-up are required.

This study did not address behavioral changes in children at school. Such tests should be performed by a standard questionnaire survey, which should be performed in the near future in a much larger cohort. However, the advantage of the present study was that we obtained data from both patients and caregivers – most mothers reported that increased frequencies of close parent–child relations during touch therapy elicited positive feelings. In conclusion, this study demonstrates that autistic behavior in school boys with ASD is treatable with a massage protocol probably due to increased salivary OT concentrations, and suggests that massage (behavioral) therapies are effective when combined with intranasal OT administration therapies.

## Conflict of Interest Statement

The authors declare that the research was conducted in the absence of any commercial or financial relationships that could be construed as a potential conflict of interest.
